# Neuropsychological considerations for long-duration deep spaceflight

**DOI:** 10.3389/fphys.2023.1146096

**Published:** 2023-05-19

**Authors:** Afik Faerman, Jonathan B. Clark, Jeffrey P. Sutton

**Affiliations:** ^1^ Department of Psychiatry and Behavioral Sciences, Stanford University, Stanford, CA, United States; ^2^ Center for Space Medicine, Baylor College of Medicine, Houston, TX, United States; ^3^ Department of Neurology, Baylor College of Medicine, Houston, TX, United States; ^4^ Translational Research Institute for Space Health, Baylor College of Medicine, Houston, TX, United States; ^5^ Department of Medicine, Baylor College of Medicine, Houston, TX, United States

**Keywords:** human spaceflight, neuropsychology, behavioral health, astronauts, human performance, cognition

## Abstract

The deep space environment far beyond low-Earth orbit (LEO) introduces multiple and simultaneous risks for the functioning and health of the central nervous system (CNS), which may impair astronauts’ performance and wellbeing. As future deep space missions to Mars, moons, or asteroids will also exceed current LEO stay durations and are estimated to require up to 3 years, we review recent evidence with contemporary and historic spaceflight case studies addressing implications for long-duration missions. To highlight the need for specific further investigations, we provide neuropsychological considerations integrating cognitive and motor functions, neuroimaging, neurological biomarkers, behavior changes, and mood and affect to construct a multifactorial profile to explain performance variability, subjective experience, and potential risks. We discuss the importance of adopting a neuropsychological approach to long-duration deep spaceflight (LDDS) missions and draw specific recommendations for future research in space neuropsychology.

## Introduction

Over the past seven decades, research in space has identified multiple implications of spaceflight on physiology and performance. As governmental and commercial space industries are growing considerably and are projected to expand even more in the following decades to come (Whealan George, 2019), prospects of long-duration deep space (LDDS) missions are currently in motion. Data collected over the years have produced a detailed and nuanced understanding of the low-Earth orbit (LEO) flight environment but limited insights regarding LDDS exposure. Given that LDDS exposure holds a set of unique and distinct mission profiles and risks, the environmental, interpersonal, and psychiatric stressors diverge from LEO missions, and thus treatment needs will likely differ as well (Smith, 2022). As such, the neuropsychological risks and countermeasures needed for LDDS missions beyond LEO are not well-defined (NASA, 2022) and are often studied separately rather than integratively.

The spaceflight environment, both in LEO and deep space, is associated with several unique conditions, some able to be simulated in Earth-based settings while others are currently not. Most notably, Earth-based space-related research has utilized analog stations to simulate isolated, confined, and extreme (ICE) environments homologous to a space station, a space vessel, or an off-Earth planetary habitat. Additionally, analog missions and Earth-based training can simulate social and teamwork-related stressors, operating equipment under limited conditions, rehearsing mission protocols, delayed communication with the Ground, and other mission-specific objectives (e.g., research experiments). Such simulations are also helpful in validating findings across different environments, settings, and populations. Conversely, some space-related conditions cannot be simulated with fidelity on Earth, such as alterations in gravity, cosmic radiation, and danger in the operating environment. Among their multisystemic effects on human health, these unique space-related conditions have been documented to impact the central nervous system in a manner that requires further consideration as LDDS missions become accessible and financially attractive. Although only a paucity of scientific studies to date examined the multifaceted implications of the deep-space environment on human cognition, behavior, and mood, anecdotal evidence and mission records allow for a better understanding of potential risks.

The National Aeronautics and Space Administration (NASA) Human Research Roadmap (HRR) recognizes that the estimation of the CNS risks due to multiple and simultaneous spaceflight hazards is hindered by a lack of relevant human data (BMed-102 (NASA, 2022b)). Further, the HRR recognizes that acute spaceflight CNS risks include neuropsychological changes which may impair astronauts’ performance and health (NASA, 2022b). Previous reviews provided immensely informative summaries and discussions of the impacts of the space environment on cognitive, behavioral, and neurological health (De la Torre, 2014; Strangman et al., 2014; Roy-O’Reilly et al., 2021; Smith, 2022). De La Torre (De la Torre, 2014) presented the importance and utility of neuropsychological considerations for space research (i.e., “space neuropsychology”) and stated that a decade ago, there was not enough evidence in this field with regard to space health. In this review, we build on previous works and present recent evidence interwoven with a neuropsychological narrative of contemporary and historic spaceflight case studies. As future deep-space missions to Mars, moons, or asteroids will inevitably exceed International Space Station (ISS) stay durations and are estimated to require up to 3 years (NASA, 2022), we focus on considerations for long-duration spaceflight. We chose this approach to overcome the main limitations of health-related research in space; nearly all data on human physiology and psychology in space come from either LEO or analog models, mostly short-term missions (likely due to the over-representation of Space Shuttle missions for the construction of the ISS, approximately 14-days (Strangman et al., 2020)), and very small sample sizes. To highlight the need for further investigations, we provide neuropsychological considerations integrating cognitive and motor functions, neuroimaging, neurological biomarkers, behavior changes, and mood and affect to construct a multifactorial profile to explain performance variability, subjective experience, and potential risks. We then highlight the importance of these considerations for the space industry and draw specific recommendations for future research.

### Structural and functional CNS alterations

Recent studies of the CNS before and after space missions indicate structural and functional alterations associated with spaceflight. Van Ombergen et al. (Van Ombergen et al., 2019) found significant increases in ventricular volume, likely due to microgravity-related reductions in cerebrospinal fluid (CSF) resorption. Consistently, prospective neuroimaging analyses by Kramer and colleagues (Kramer et al., 2020) revealed that long-duration spaceflights were associated with alterations in CSF hydrodynamics, as well as deformation of the pituitary gland. Additionally, Jillings et al. (Jillings et al., 2020) observed increases in cerebellar white matter after spaceflight. These findings also denoted a long-term (potentially permanent) structural impact as these volumetric increases remained significant even after a 7-month (Van Ombergen et al., 2019; Jillings et al., 2020) and 1-year follow-ups (Kramer et al., 2020). Most recently, Doroshin et al. (Doroshin et al., 2022) analyzed microstructural tractography changes in 12 cosmonauts after an average 6-month stay aboard the ISS and observed significant changes in multiple large white matter tracts associated with sensorimotor processes. In partial alignment with the stability of the volumetric changes observed by Jillings et al. (Jillings et al., 2020), some of the observed changes remained present at the 7-month follow-up, while others (e.g., corticostriatal tracts and corpus callosum) recovered back to baseline. Functionally, significant pre-to post-spaceflight changes in functional connectivity of visual with visuomotor and visual-frontal structures were associated with spatial working memory (Salazar et al., 2022), likely representing spaceflight-related disruptive impacts and compensatory shifts in the brain. Other studies also found decreased functional connectivity between the cerebellum and networks that play a role in vestibular, visual, motor, and sensory processing (Demertzi et al., 2016; Pechenkova et al., 2019). For a comprehensive review of brain alterations during spaceflight, see Roy-O’Reilly et al. (Roy-O’Reilly et al., 2021).

Some of the structural changes likely represent adaptive neuroplasticity in brain tissues (Jillings et al., 2020). However, spaceflight-related changes in the brain may reflect an overall risk for immediate and long-term damage, as indicated by both biomarker and performance-based evidence. Long-duration spaceflight was associated with significant increases in neurofilament light chain (NfL), a marker for axonal disintegration, and glial fibrillary acidic protein (GFAP), a marker for astrocytic activation (zu Eulenburg et al., 2021). Moreover, zu Eulenburg et al. (zu Eulenburg et al., 2021) found post-flight increases in the accumulation of amyloid-β peptides, Aβ40 and Aβ42, critical proteins in the neurodegenerative processes of Alzheimer’s disease pathologies (Qiu et al., 2015). Post-flight mice models showed immunohistochemical evidence of damage to the blood-brain barrier, alterations in neurovascular and neuronal structure, reduced mitochondrial function and decreased overall brain metabolism (Mao et al., 2020). These elevated markers of brain tissue damage could represent reparatory processes due to microgravity-related intracranial hypertension (zu Eulenburg et al., 2021; Michael and Marshall-Bowman, 2015). Spaceflight-related increases in intracranial pressure (ICP) is a theoretical symptom that has largely gained acceptance as a recognized clinical phenomenon following supportive indirect evidence (e.g., increased ICP by lumbar puncture in symptomatic astronauts upon return to gravity; also, spaceflight-associated visual pathologies have a similar presentation to cases of known ICP on Earth), despite lack of direct evidence of ICP in space (Michael and Marshall-Bowman, 2015). Beyond increased risk for tissue damage, long-term intracranial hypertension is a risk factor for hemorrhage, infection, postlumbar puncture headache, and spinal cord injury (Barr, 2014; Michael and Marshall-Bowman, 2015). Although stroke poses the most immediate danger to health and performance, long-term changes in brain vasculature could augment neurocognitive deficits due to vascular reasons (e.g., “vascular dementia”), leading to clinically meaningful declines in cognitive performance, mood, and health (Bir et al., 2021).

Upward redistribution of CSF (Roy-O’Reilly et al., 2021) could interrupt systemic processes responsible for both short- and long-term cognitive health and performance. For example, impaired CSF flow might impede glymphatic system function. A recently discovered CNS waste clearance system, the glymphatic system utilizes astroglial-based perivascular “tunnels” to mobilize soluble proteins and metabolites from interstitial space to perivenous drainage pathways (Jessen et al., 2015). As such, suboptimal functioning of glymphatic clearance could progressively lead to interruption of neuronal health and the accelerated accumulation of protein waste associated with neurodegenerative diseases such as Alzheimer’s (amyloid-β) and Parkinson’s (α-synuclein). Post-flight elevations in GFAP and amyloid-β (zu Eulenburg et al., 2021) support this hypothesis. Indeed, a recent study by Barisano et al. (Barisano et al., 2022) analyzed volumetric alterations in perivascular spaces (PVS) of international space screws after six monoths on the ISS, and found increase in PVS volumes in the basal ganglia and white matter post-spaceflight. Such PVS changes could lead to morphplogical changes of glymphatic pathways and impair glymphatic clearance, as seen in aging (Kress et al., 2014). White matter PVS volume changes also correlated with enlargement of the lateral ventricles and shrinkage of the subarachnoid space at the vertex. Intrestingly, despite exposure to the same ISS environment for the same duration, NASA astronauts showed greater white matter PVS changes than Roscosmos cosmonauts, attributed by the authors to brain fluid redistribution due to different countermeasures and resistance exercise routines (Barisano et al., 2022). Furthremore, NASA astronauts who developed spaceflight-associated neuroocular syndrome (SANS), swelling in the back of the eye that impacts visual acuity in about 60% of astronauts (Lee et al., 2017), had greater white matter PVS volume changes (Barisano et al., 2022). A detailed summary of relevant molecular, neurochemical, and neurobiological evidence from animal models and suggestions for further rodent and primate studies can be found in a recent review by Desai et al. (Desai et al., 2022).

### Radiation: the current frontier

The majority of current NASA-funded space-related CNS research is focused on the adverse effects and possible acute risks of galactic cosmic rays (GCR; highly energetic, fully ionized atomic nuclei) and solar particle events (SPE; Sun-emitted protons which are accelerated by a solar flare or coronal mass ejection) (NASA, 2022b; NASA, 2022). Ionizing radiation in the space environment may increase the risk of degeneration of bodily tissue, carcinogenesis, and acute radiation syndromes (Mi and Norman, 2020). Specific concerns were raised regarding the acute and late effects of ionizing radiation in space on the CNS, particularly in the context of LDDS missions (Mi and Norman, 2020; Pariset et al., 2021). Irradiation effects on the CNS include DNA damage, necrosis, oxidative stress, and systemic inflammation (Pariset et al., 2021). Furthermore, experimental studies in mice demonstrated that CA1 pyramidal neurons that were neutron-irradiated are less excitable (Acharya et al., 2019), consistent with previous findings showing that GCR exposure leads to reductions in neurotransmitter expression (Carr et al., 2018) and that exposed neurons in the hippocampus and perirhinal cortex undergo membrane hyperpolarization (Sokolova et al., 2015; Parihar et al., 2018) and structural alterations in dendritic spines (Carr et al., 2018). Similarly, rat models found irradiation to be associated with deficits in dopaminergic pathways, which were later correlated with attentional impairments (Cucinotta et al., 2014). Moreover, two studies suggested ionizing radiation exposure might accelerate Alzheimer’s disease progression in mice (Vlkolinsky et al., 2010; Cherry et al., 2012; Cucinotta et al., 2014).

While the vast majority of relevant research has been done in rodent models, with minimal and indirect evidence in humans (George et al., 2010; Cucinotta et al., 2014; Garrett-Bakelman et al., 2019), sufficient evidence suggests that radiation dose and quality have differential detrimental effects on cognitive performance (Cacao and Cucinotta, 2019) and are linked to the emergence of distress behaviors (Acharya et al., 2019; Pariset et al., 2021). Although no direct evidence links radiation damage to cognitive sequelae in space crews (an environment with greater risk for radiation-related biological damage (Straume, 2018)), evidence from radiation therapy links neurodegenerative conditions and neurobehavioral symptoms following treatment (Crossen et al., 1994; Peper et al., 2000; Klein et al., 2002). However, the uncertainty around functional and safety threshold for exposure hinders necessary decision-making for LDDS missions such as to Mars (Straume, 2018). These led several authors and space agencies to highlight the incremental risk of LDDS radiation exposures to human cognition, astronaut acute and long-term neurological and emotional wellbeing, and mission safety.

It is important to note that evidence from radiation therapy patients is likely heavily confounded by the disease being treated. Although radiation-related effects have been studied in otherwise healthy populations, most studies mainly focused on health outcomes and not pre/post-exposure changes in performance or biomarkers. For example, recent literature suggests that long-haul high-altitude airline crew and passengers are exposed to radiation levels that likely pose a variety of health risks, specifically the risks of cancers, including brain tumors (Olumuyiwa, 2020). Similarly, analyses of cohorts exposed to the Chernobyl accident found inconsistent results but were heavily confounded by methodological pitfalls (Cucinotta et al., 2014). However, recent meta-analyses indeed found exposure to low-to-moderate doses of ionizing radiation to be associated with cardiovascular disease incidence and mortality (Lopes et al., 2022a), but not risk for developing CNS tumors (Lopes et al., 2022b). Interestingly, a linear relationship between Parkinson’s disease incidence and cumulative exposure to gamma radiation was observed in employees of the Mayak Production Association, one of Russia’s largest nuclear facilities (Azizova et al., 2020). This finding was later supported by the Million Person Study of American workers and veterans who were exposed to radiation from 1939, suggesting a potential dose-response relationship with Parkinson’s disease incidence in at least one of the cohorts (Boice et al., 2022; Zablotska et al., 2022). Nevertheless, these data are entirely observational and likely are confounded by a variety of interindividual differences, some with potential impact on radiation effect on the CNS (Cucinotta et al., 2014).

The complex network of ionizing radiation impact on the CNS requires multifaceted preventative countermeasures and reparative treatments. In a recent review, Pariset et al. (Pariset et al., 2021) summarized various approaches currently discussed to mitigate radiation-related CNS impairments. Current approaches target one of five main mechanisms: DNA damage, inflammation, reactive oxygen species, cell survival, and tissue repair. In their analysis, Pariset et al. (Pariset et al., 2021) emphasize that, for countermeasures to be practical and efficient, they would have to be administered peripherally, with minimal need for repeated or continuous administration and little to no side effects.

### Sleep and circadian health

Sleep disturbance, among the most common complaints by astronauts (Barger et al., 2014), has been robustly linked to specific deficiencies in cognitive performance on Earth (Lim and Dinges, 2010). Unsurprisingly, sleep remains impactful on cognition during spaceflight as well (Wu et al., 2018). An additional consequence of inadequate sleep (Darwent et al., 2015), excessive daytime fatigue, is a documented factor in decreased astronaut productivity and performance (Eddy et al., 1998). Although acute sleep disruption may be more impactful on basic attentional skills than on complex cognitive tasks and executive functions (Lim and Dinges, 2010; Wickens et al., 2015), prolonged disturbances in sleep are likely to impact executive functions and memory, as seen in individuals with insomnia (Fortier-Brochu et al., 2012). Indeed, several historical incidents highlight the importance of sleep and fatigue for mission safety and success. Post-incident analyses indicated critical cognitive and psychosocial factors that led to the 1997 collision of the Russian supply shuttle *Progress 234* with the *Mir* space station. The collision ruptured Mir’s pressure hull, and the station was almost evacuated after developing an uncontrolled attitude drift. Cosmonaut Vasili Tsibliyev, the Progress 234 commander, reported poor sleep 2 weeks before the crash and having only 2 days of rest in the 4 months leading to the crash (Ellis, 2000). Several cognitive factors played a role in the collision, including inaccuracies in visuospatial (e.g., lack of detection of the Mir using the Toru docking monitor) and sensorimotor (e.g., in operating the attitude thrusters) performance and suboptimal decision making (e.g., the shutdown of the Kurs radar) (Ellis, 2000). Other psychosocial factors contributed, such as elevated stress (e.g., Tsibliyev failed his previous Mir docking with Progress-233 (Ellis, 2000) and was likely stressed to succeed docking with Progress-234) and the suboptimal relationship between the cosmonauts and Russian Mission Control (Oberg, 1998). Less than a month later, during a spacewalk training exercise to reconnect power cables to three solar arrays undamaged from the collision, Mir-23 Flight Engineer Aleksandr Lazutkin disconnected a wrong power cable routing power and data to the attitude control computer (NASA, 1986). As a result, the Mir lost orientation to the Sun and had a total power shutdown to the station. Furthermore, long-duration spaceflight poses greater concerns regarding the detrimental effects of inadequate sleep on performance and health. After studying astronaut Jerry Linenger’s circadian markers across 112 days on the Mir, Monk et al. (Monk et al., 2001) concluded that spaceflights longer than 100 days might lead to an accumulative failure of the human endogenous circadian pacemaker to drive a 24-h circadian rhythm. Consequently, such circadian deviation can contribute to sleep problems, particularly when forcing a 24-h schedule (Monk et al., 2001; Guo et al., 2014). Anecdotally, cognitive performance in the last third of the mission was notable for increased speed (potentially due to practice effects) and reduced accuracy (Monk et al., 2001). Indeed, NASA’s HRR recognized sleep as a major area of interest (Gregory, 2016); however, while operational impact and long-term health risks of sleep issues in LEO, lunar orbit, and lunar surface are largely accepted with optimization strategies, both aspects are yet to be mitigated for a LDDS Mars mission (Gregory, 2016).

Beyond direct and indirect impacts on performance, prolonged sleep and circadian deficits also pose risks to CNS health. Self-reported disrupted sleep is associated with a substantial increase in risk for Alzheimer’s disease and other dementia (Benedict et al., 2020). Additionally, acute sleep deprivation leads to elevated levels of CSF of tau (Holth et al., 2019), and disrupted sleep due to sleep apnea was associated with amyloid-β. Slow-wave sleep appears to decrease with Alzheimer’s disease progression, mainly tauopathy (Lucey et al., 2019), potentially reflecting a decrease in glymphatic clearance of amyloid-β and tau (Cedernaes et al., 2017). Consistently, glymphatic clearance rates in rodent models appear to be twice faster during sleep than during wake (Xie et al., 2013), highlightinh the Adequate sleep is particularly crucial with regards to glymphatic clearance (Mendelsohn and Larrick, 2013), Although dementias manifest mostly in older adults, a recent study found acute sleep loss to associate with elevated tau levels in the blood, potentially suggesting detrimental effects on brain health even in young adults (Benedict et al., 2020). Consistently, in mice, chronic short sleep is associated with reductions in CA1 pyramidal neuron quantity and volume, impaired spatial memory, and increased amyloid-β and tau (Owen et al., 2021). Aside from sleep-related risk, a growing body of evidence links circadian disruptions to a risk of developing or exacerbating neurodegenerative processes, such as accelerated temporal lobe atrophy, increased CSF biomarkers of proteinopathy, and increased risk of mild cognitive impairment in delayed activity rhythms (Cedernaes et al., 2017; Nassan and Videnovic, 2022). Furthermore, mice models of circadian disruption indicated impaired functional connectivity, greater neuronal oxidative stress, and increased permeability of the blood-brain (likely due to governing role of an endogenous circadian rhythm on the barrier transporter functions) (Cedernaes et al., 2017; Cuddapah et al., 2019). Additionally, disruptions to sleep-related clearance of perivascular waste can interfere with the movement of molecules across and along the blood-brain barrier leading to increased permeability and barrier breakdown (Cedernaes et al., 2017; Cuddapah et al., 2019). In LDDS the risks of sleep and circadian disruptions are not isolated from radiation and gravity effects. As such, the potential accumulation of stress on the CNS is particularly salient for LDDS missions, and the need to formalize risks and develop effective countermeasures for prolonged disturbed or inadequate sleep is high.

## Clinical and performance implications

### Cognitive performance in space

Although no evidence to date indicates an increased prevalence of neurodegenerative disease in humans who were exposed to spaceflight environments, current literature suggests that longer spaceflights pose considerable neurocognitive risks. Failures of attention or task planning can and have put the lives of astronauts and cosmonauts at risk. For example, during the 96-days-long Salyut 6 EO-1 mission in 1977, mission commander Yuri Romanenko forgot to attach his safety cord while preparing for a spacewalk. He was pushed outside, and flight engineer Gregory Grechko managed to grab Romanenko’s safety cord with one hand and pull him back into the airlock (JSTOR, 1978; Harland, 2007).

Tracking the independent impact of long-duration spaceflight-related brain alterations on cognition is a difficult task, particularly when cognitive functioning is commonly confounded by sleep, fatigue, and other contextual factors. For example, before the implementation of modern computerized batteries, neuropsychological testing during spaceflight was considerably limited (Strangman et al., 2020). Older measures were conceptualized through a brain injury paradigm and lacked sensitivity in normal brain functioning and above-average populations such as astronauts (Strangman et al., 2020). Furthermore, most tests were not designed for recurring administration, and practice effects were notable across many studies (Strangman et al., 2014). As such, rather than highlighting consistent cognitive domains sensitive to the spaceflight environment, data suggests intraindividual alterations in cognitive performance (Strangman et al., 2014). For example, the NASA Twin Study (Garrett-Bakelman et al., 2019) compared cognitive performance, using the Cognition (Basner et al., 2015) computerized test battery, over 1 year between inflight and Earth-based identical twin astronauts. Cognition was developed for astronauts and was recently found to have good acceptability in astronaut and astronaut-surrogate cohorts across various mission settings and durations (Casario et al., 2022). From early to late flight, the inflight astronaut had significant reductions in visuospatial distinction (Abstract Matching), visuomotor speed (Digit Symbol Substitution Task), and in the Emotion Recognition Task (Garrett-Bakelman et al., 2019). Interestingly, compared to the Earth-based control astronaut, the inflight astronaut demonstrated decreases in visual learning and matching and greater risk-taking (Balloon Analog Risk Test) (Garrett-Bakelman et al., 2019).

A multitude of factors can impact cognitive performance during spaceflight, including living in an ICE environment (Connaboy et al., 2020) or unexpected events such as dehydration (Wittbrodt and Millard-Stafford, 2018), carbon dioxide (CO2) spikes (Scully et al., 2019), exposure to toxic gases and substances (Strangman et al., 2020), and noise (Szalma and Hancock, 2011). In their review of cognitive performance in spaceflight, Strangman et al. (Strangman et al., 2014) reported modest evidence in spaceflights >90 days (31 subjects across seven studies) for impairments in attention, speed of visuomotor tasks, and time perception (underestimation), while performance on mental rotation of visual objects was intact or had minimal improvement. Interestingly, they also found spaceflight to be associated with increases in the variability of cognitive performance (Strangman et al., 2014). It is important to note that although these findings are classified for “very long-duration” spaceflights, future missions planned for years might involve risks that are not well-captured in shorter durations. In a more recent study, Roberts et al. (Roberts et al., 2019) demonstrated that spaceflight-related brain changes are associated with alterations in cognitive and motor performance and progress based on mission duration. The authors tested relationships between structural brain changes and neuropsychological performance in 12 long-duration astronauts on the ISS using the Spaceflight Cognitive Assessment Tool for Windows (WinSCAT) and with motor performance in eight astronauts using the Functional Task Test. Spaceflight-related changes in cognitive performance were significant for reduced accuracy on a processing-speed and learning task (Code Substitution; CDS), but faster reaction times on both the CDS and a measure of sustained attention (Continuous Performance Test; CPT), interpreted by the authors as a likely consequence of practice effects (Roberts et al., 2019). Structural post-flight changes in the bilateral optic radiations (right more than left) and splenium (the posterior end of the corpus callosum) were negatively associated with a change in CPT reaction time. Additionally, post-flight ventricular enlargement had a strong negative association with CPT reaction time. These volumetric changes were interpreted as a compensatory process that allowed the preservation of intact performance (Roberts et al., 2019). Interestingly, post-flight ventricular enlargement was also negatively correlated with CDS accuracy, but not after correcting multiple comparisons. Lastly, changes in the right lower extremity primary motor area (or midcingulate) were significantly associated with the completion time of the Seated Egress and Walk Test, a complex motor task involving an obstacle course. Importantly, the extent of ventricular changes was negatively correlated with age, indicating that younger astronauts may experience greater CNS alteration (Roberts et al., 2019). The findings by Roberts et al. (Roberts et al., 2019) might also indicate differences between the space environment and Earth-based ICE environment, as a recent study in ICE environment analogs (Connaboy et al., 2020) found that almost all WinSCAT measures improve over 5-month missions.

Accumulating evidence robustly links microgravity with sensorimotor and visuospatial alterations. Conceptual frameworks have suggested that when vestibular inputs are disrupted, the CNS rapidly adapts by updating internal prediction models of sensory implication on motion and proprioception and up-weighting non-vestibular information that appears more reliable (Clément and Ngo-Anh, 2013; Carriot et al., 2015). For example, gravity alterations interfere with visual perception stability (Clément and Demel, 2012), and both short (e.g., 2 weeks) and prolonged (e.g., 6 months) exposure to microgravity appears to disrupt vestibular inputs and processing, leading to declines in postural control, balance, and mobility (Wood et al., 2015; Ozdemir et al., 2018; Tays et al., 2021). However, these effects appear to recover back to baseline within two to 4 weeks (Wood et al., 2015; Ozdemir et al., 2018; Tays et al., 2021). Beyond the impacts on sensorimotor performance, gravity-related alterations of vestibular signals can impact spatial cognitive functions such as mental imagery, visuospatial reasoning, and number processing (Mast et al., 2014). When such vestibular disruptions are present, other multisensory stimuli can aid in representational stability. For example, Tays et al. (Tays et al., 2021) showed a trend toward better in-flight performance on the cube rotation task when crewmembers were able to anchor themselves to the floor using foot loops. Using a parabolic flight paradigm, Salatino et al. (Salatino et al., 2021) found that zero gravity enhances bottom-up visuospatial attention while weakening voluntary sustained attention. These findings emphasize the possibility that bottom-up processes drive some of the cognitive alterations observed in space crews (e.g., faster reaction times). Furthermore, the vestibular system is responsible for several multisensory components of body representation and, as such, plays a role in neuropsychological processes such as psychomotor performance, pain, and orientation and can influence mood and behavioral health (Mast et al., 2014), potentially portraying vestibular dysfunctions as a risk factor for psychiatric symptomatology.

Interestingly, in a bidirectional manner, top-down processes (e.g., mental imagery) can modulate the perception of and response to vestibular stimuli. Consistently, maladaptive top-down processing may negatively impact vestibular functioning, as there appears to be high comorbidity of psychiatric symptoms and vestibular dysfunctions (Mast et al., 2014). In studies funded by the Italian Space Agency, individuals with a stronger trait-like ability to facilitate experiential changes in response to verbal suggestions (i.e., hypnotizability) showed lesser dependence on sensory inputs, better locomotion accuracy, and greater performance benefits from practice (Menzocchi et al., 2009; Menzocchi et al., 2010), suggesting that psychological traits may moderate the effects of vestibular disruptions on performance and other related systems. Overall, vestibular alterations in space may be intrinsically related to cognitive and behavioral health, and addressing developing relevant countermeasures for LDDS missions is needed not only for the in-flight duration but also for the adaptation periods when gravity is reintroduced (e.g., planetary mission) (Clément and Ngo-Anh, 2013).

Space adaptation syndrome (SAS; also called space motion sickness (Strangman et al., 2020)) and complaints about a subjective deterioration of attention and the ability to think clearly (i.e., “mental viscosity,” “space fog,” or “space stupids” (White et al., 2016)) might represent the subjective manifestation of neurocognitive and vestibular adjustments to the space environment (De la Torre, 2014). It is estimated that approximately 70% of space travelers experience SAS (De la Torre, 2014), but these are mostly transient phenomena that resolve after a few days in the case of space fog (Welch et al., 2009) and days to weeks for SAS (Strangman et al., 2020), thereby aligning with the arguments for a neurocognitive adaptation period to spaceflight (Roberts et al., 2019; Roy-O’Reilly et al., 2021). Russian psychologists and flight surgeons identified a long-duration spaceflight syndrome characterized as a “nervous or mental weakness,” and its symptoms include physical or emotional tiredness and fatigue, loss of strength and hypoactivity, attention and memory deficits, sleep disturbance, irritability, volatile mood, poor appetite, and low sensation threshold (Petrovsky and Yaroshevsky, 1987; Kanas and Manzey, 2008). The syndrome was termed “asthenia” (a milder form of neurasthenia (Kanas and Manzey, 2008), F48.8 in ICD-10) and was argued to be somatic in nature and to develop following “excessive mental or physical strain, prolonged negative emotional experiences, or conflict.” (Petrovsky and Yaroshevsky, 1987) There is a paucity of evidence regarding asthenia in space, and findings largely fail to consistently support it (Kanas and Manzey, 2008).

### Mood and behavior

Alongside potential alterations in basic and high-order cognitive processes, the individual and compounded effects of the demanding ICE environment, sleep disturbances, structural and functional brain changes, and radiation exposure inevitably impact mood and behavior. Indeed, concerns about psychological problems in LDDS exploration were raised in the early phases of the space race by both NASA (e.g., Werner von Braun in 1954) and ROSCOSMOS (e.g., by cosmonaut Valery Ryumin in 1980) (Stuster et al., 2020).

Besides the fact that space missions are long-awaited by the crew, which could increase mission-related stress and the strive for mission success, the space-related CNS-compromising factors reviewed above may have implications on space crews’ mood and behavior. Specifically, alterations in emotion regulation can have direct implications on astronauts’ wellbeing, performance, and mission safety and success. The superior temporal gyrus and the supplementary motor area, brain regions that have been found in meta-analytic evidence to be involved in emotion regulation (Kohn et al., 2014; Morawetz et al., 2017), undergo significant morphological changes during spaceflight (Koppelmans et al., 2016; Van Ombergen et al., 2019; Hupfeld et al., 2020). Attentional abilities, which may be impaired in long-duration missions, play a central role in emotion regulation, particularly in changing the focus of attention to and from emotionally salient stimuli (i.e., attentional deployment) (Turnbull and Salas, 2021). Following a 169-day-long mission, a cosmonaut experienced decreased intrinsic functional connectivity in the right insula (Demertzi et al., 2016), a region that is involved in emotion regulation independent from strategy, with greater involvement in attention-related emotion regulation (Morawetz et al., 2017). Sleep is also a key factor in emotion regulation. Inadequate sleep is linked to increased negative and reduced positive emotions and can directly and indirectly (e.g., via motivation and goal-reward evaluation) interfere with cognitive regulatory processes of emotion (Palmer and Alfano, 2017).

There have been several cases of disproportional emotional responses and consequent behaviors in long-duration LEO space missions. For example, STS-51B payload specialist Taylor Wang had an experiment delayed due to a faulty instrument, but NASA denied him the opportunity to repair it. In recounting his experience, he described that, out of desperation, he said that if he is not given a chance to repair his instrument and repeat his experiment, he is “not coming back” (Reichhardt, 2002). This statement reportedly led NASA to assign a psychologist to interview the STS-51B crew members about Wang’s mental wellbeing. Mission commander, astronaut Bob Overmyer, indicated that Wang was depressed over the failure of his experiment (Reichhardt, 2002). Similarly, after receiving the news of his mother’s passing, Mir 18 Commander Vladimir Dezhurov separated from the crew and secluded himself in a module for days (Dudley-Rowley, 2006). Despite the emotional toll of losing a parent, extreme response to grief may be influenced by compromised emotion regulation processes. Cosmonaut Valery Ryumin contemplated that “all one needs to effect a murder is to lock two men in a cabin eighteen feet by twenty feet and keep them there for 2 months.” (Oberg, 1981) And, indeed, during his 211-day-long mission on the Salyut 7, cosmonaut Valentine Lebedev estimated that 30% of time-in-mission was spent in interpersonal conflict (Palinkas, 2001) and that physical and verbal mannerisms became sources of interpersonal tension between him and his crewmember, cosmonaut Anatoly Brezevoy (Kanas et al., 2020). Consistently, in a 520-day-long analog mission, as part of the MARS 500 study, two crewmembers with the highest stress and exhaustion levels accounted for 85% of the conflicts (Basner et al., 2014). Notably, one of these individuals developed insomnia symptoms with chronic partial sleep deprivation, daytime tiredness, and frequent impairments to alertness (Basner et al., 2014). However, crew-mission control conflicts were five times more prevalent than within-crew interpersonal conflicts (Basner et al., 2014).

Potentially necessary for in-flight maintenance during LDDS missions, spacewalk itself can bring a wide spectrum of emotional experiences. For example, after having been ordered to climb back into the capsule at the completion of the first American spacewalk, Gemini 3 Astronaut Ed White stated it was “the saddest moment of my life.” (MSFC, 2009) On the other hand of the spectrum, during his 132-days-long mission between STS-81 and STS-84, Linenger described the feeling of spacewalking as “difficult to discount the sensation that you are moving away, alone, detached … You are hanging to the thinnest limb of the tallest tree in the wind. The tree is falling” (NASA, 1997).

Compounding on the space-related compromise to emotion regulation abilities, LDDS missions are going to introduce considerable differences from lunar or LEO missions (e.g., ISS) due to the length of travel. While LEO missions often include a busy schedule to maximize research and task output in a given timeframe, LDDS missions are likely to become increasingly autonomous (NASA, 2022b). As such, LDDS inevitably requires a different model of psychological wellbeing, addressing the lack of live ground support. Given the different nature of LDDS missions from ongoing space missions, this new psychological model will lean mostly on findings from Earth-based analog missions, as well as insights from homologous environments. Remote polar and space missions have identified time effects on psychological functioning. Specifically, subjective reports indicate an increase in emotional difficulties and interpersonal problems after the halfway point of the mission (aptly termed the “third-quarter phenomenon”) (Kanas et al., 2020; Stuster et al., 2020). Although it is possible that an emotional letdown follows the realization of the need to spend an equivalent amount of time before the return home (Kanas et al., 2020), it is also possible that space-related brain changes accumulate over time and show greater effects on mood and behavior after a substantial amount of time has passed. However, this phenomenon did not appear to have a statistically significant effect on mood or interpersonal cohesion in either the Mir or ISS, possibly due to psychosocial support from crewmembers who had increased or no change in emotional status after the halfpoint (Kanas et al., 2020).

Another potential for emotional compromise in LDDS missions stems from the distance from Earth. Across studies done in both the ISS and remote duty stations, conflicts with ground control proved a major contributor to space crews’ stress (Stuster et al., 2020). This issue could prove substantially more challenging as communications delays between the crew and Earth-based control increase with distance from Earth (Kanas et al., 2020), as confirmed by the MARS 500 analog missions (although the impact was mainly due to the presence of delays rather than their length) (Ushakov et al., 2014). In a survey about the emotional reaction to being in space, all 39 astronauts and cosmonauts endorsed at least some level of positive change (Ihle et al., 2006). A content analysis revealed one significant factor in producing such a change: perceptions of Earth. Of the different items under this factor, the highest impact came from an increasing appreciation of the Earth’s beauty. This item, together with two other items about realizing how much one cares for the Earth and appreciates its fragility, were significantly related to endorsing increases in environmental involvement after returning to Earth. This psychological phenomenon was previously termed The Overview Effect by philosopher Frank White (White, 1998). Viewing the Earth appears to be emotionally salient to astronauts. Within the first 3 years of the ISS, almost 200,000 photos of Earth were taken, with almost 85% being crew-initiated (Yaden et al., 2016). Consistently, anecdotal evidence suggests that denying crew the opportunity to view the Earth may exacerbate conflicts (e.g., Skylab IV) (Yaden et al., 2016). In LDDS missions, however, crewmembers will likely lose direct visual contact with the Earth. This may have implications for crewmembers’ sense of safety (a reminder of the distance from help in case of emergency), belonging, motivation, and wellbeing. For further reading about behavioral health risks and supportive measures in LDDS missions, see a scoping review by Smith (Smith, 2022).

## Future research and clinical directions

Given the multicomponent complexity of the effects of deep-space environments on the CNS, an integrated approach to identifying optimal measures, interventions, and countermeasures is warranted. Here, we apply a neuropsychological approach, taking together evidence from neuroimaging, cognitive performance, and behavioral functioning based on recently published evidence and historical accounts. This approach is consistent with NASA’s HRR design, identifying a need for practical tools for monitoring and measuring changes in cognitive and behavioral health and performance (NASA, 2022b). Most importantly, the interactions and combined effects of prolonged exposures to gravity alterations, radiation, and sleep and circadian disruptions during LDDS missions, and the interrelatedness between cognitive and behavioral health highlight a clear need for assessment tools that can account for these combined effects, rather than evaluating them separately. Such tools should be developed for flight surgeons and operational psychologists to use in-mission and utilize both objective assessments and crewmembers’ subjective reports. Furthermore, developing such integrated methods could aid in optimal astronaut selection and training and personalize communication, expectation, and interventions (NASA, 2022b; Smith, 2022). First and foremost, there is a crucial uncertainty about whether gravity alterations, radiation exposure, and potential sleep and circadian disruptions in LDDS missions pose a risk for acute neurological damage such as stroke or tissue atrophy due to the possibility of intracranial hypertension. This is a priority, as identifying the severity of such risk would advise the implementation of preventative strategies to reduce it. Moreover, evidence suggests that prolonged exposure to radiation (particularly in high doses as expected in LDDS missions) and sleep loss could accelerate neurodegenerative processes. At this point, not only is there insufficient data to evaluate the likelihood of this risk in humans but no estimate of the speed of neurodegenerative progression and whether it is reversible or permanent (particularly in light of transient and reversible structural changes observed in 6-month missions in LEO). Furthermore, it is yet unclear whether these changes underlie the observed alterations in cognitive performance in space, although current data suggests only a moderate impact in LEO missions.

Historically, psychological screening has proved insightful in several anecdotal cases. For example, both American and Russian psychologists expressed concerns regarding astronaut John Blaha’s readiness for a long-duration mission on the Mir. And indeed, Blaha was later reported to experience depression and anger bursts during the mission (Dudley-Rowley, 2006). Given advancements in measures and greater access to more comprehensive sets of behavioral, cognitive, and neural data, psychological screening should adopt a neuropsychological approach and integrate the multitude of information sources to draw more informed conclusions and make more accurate predictions about astronauts’ strengths, weaknesses, and potential risks. This is ever more relevant for LDDS missions due to the increased CNS risk involved. While such approaches currently exist in clinical contexts, further development of a neuropsychological process for astronauts and other high-performing individuals is needed. Such development should expand available normative data, design optimal assessment protocols, delineate a mechanism to integrate all available data points, and provide a detailed personalized output for agencies to work from.

Alongside behavioral and neurocognitive evaluations, sleep assessments before, during, and after missions are essential. Apart from sleep duration, a central outcome variable for sleep health, monitoring the subjective quality of sleep may be crucial for detecting sleep-related deficits to emotion regulation (Palmer and Alfano, 2017). Additionally, people vary substantially, in a trait-like manner, in the extent to which their cognitive and behavioral performance is impacted by inadequate sleep. Similar trait-like factors could also be identified for susceptibility to acute or late cognitive and behavioral detriments, as well as a propensity for CNS damage in the space environment (e.g., synaptic dysfunction, impaired neurogenesis, neurodegeneration, proteinopathies, neuroinflammation) (NASA, 2022b). At this point, no such metrics exist for LDDS missions, nor do validated norms for many of the currently measurable variables (NASA, 2022b). Identifying correlates of this and other trait-like factors could reduce the burden and urgency of managing acute risks while ensuring crewmembers’ health and wellbeing. Furthermore, although circadian-based interventions, such as light therapy, scheduled physical activity, and melatonin supplementation, have been tested in LEO missions, the development and validation of new treatments for LDDS could help maintain circadian homeostasis and possibly combat the risk for accelerated neurodegenerative processes (Nassan and Videnovic, 2022).

However, even after accounting for individual abilities to perform under stress and duress, space agencies seek validated performance outcome limits (POLs) and Permissible Exposure Limits (PELs) (NASA, 2022b). POLs and PELs are intertwined, as PELs should be determined based on POLs and health measures, while PELs should be taken in the context of environmental factors. For example, alongside research into the biological impacts of radiation exposure, further research is needed into the cognitive and behavioral implications in humans (NASA, 2022). Although sufficient evidence has been accumulated to safely assume that GCR and SPE negatively impact the CNS, identifying their effect on human performance (POL) will help set exposure boundaries (PEL). Similarly, a better understanding of the implications of space-related CNS structural and functional changes on human performance, health, and wellbeing is acutely needed. Further research is needed into the optimal operational definition and classification of POLs and PELs, given the available and constantly growing data pools. From an integrative standpoint, there is an operational need for the development of PELs of inadequate sleep before sleep-related cognitive and behavioral impairment appears, as well as PELs of negative moods before a negative impact on sleep and cognitive performance is observed.

Lastly, adopting and promoting a neuropsychological approach to LDDS missions (i.e., “space neuropsychology” (De la Torre, 2014)) could accelerate Earth-based brain health research and development. For example, the design and development of direct and embedded tools to evaluate real-time cognitive performance could be used for or adapted to clinical evaluations or operational applications such as military, humanitarian, or emergency service organizations. For example, the Cognition test battery (Basner et al., 2015), which was developed for astronauts, has been utilized for several Earth-based investigations (Beckner et al., 2021; Abeln et al., 2022; Makowski et al., 2022; Tait et al., 2022). Moreover, space-oriented investigations are in progress to test whether introducing artificial gravity during long bouts of simulated microgravity (bed rest) can counteract the development of neurocognitive declines (NASA, 2023aa) or structural and functional alterations in the human brain (NASA, 2023ab). Furthermore, repurposing drugs currently used for CNS disorders may prove useful for preventing and, in some cases, repairing CNS damage in space. In turn, this could increase resources allocated to advancing drug development and off-label clinical research. Similarly, as radiation-related CNS damage is understood as a form of neurological damage or accelerated aging (Pariset et al., 2021), the development of radiation countermeasures may prove beneficial for aging and neurological disorders on Earth.

## Conclusion

In this review, we utilize a neuropsychological approach to integrating clinical, performance, neuroimaging, and biomarker evidence from spaceflight studies to better characterize the potential risks of LDDS missions. Although the current literature draws heavily on LEO and relatively short-duration missions, we highlight potential risks for long-term exposure to the space, and particularly deep space, environment. We argue for a need for specific Earth- and space-based investigations and call for more research to adopt a neuropsychological approach to LDDS missions.
